# 812. Peripheral Vascular Disease Worsens Outcomes in Lower Extremity Burns: A National Database Analysis

**DOI:** 10.1093/jbcr/irag033.251

**Published:** 2026-04-06

**Authors:** Mare G Kaulakis, Hilary Liu, Daniel Najafali, Sarah M Tepe, Christopher J Fedor, Kurt Stahlfeld, Alain C Corcos, Francesco M Egro

**Affiliations:** University of Pittsburgh Medical Center, Mercy Burn Center, Pittsburgh, PA; University of Pittsburgh Medical Center, Pittsburgh, PA; Carle Illinois College of Medicine, Champaign, IL; University of Pittsburgh Medical Center, Pittsburgh, PA; University of Pittsburgh Medical Center, Department of Plastic Surgery, Pittsburgh, PA; University of Pittsburgh Medical Center, Mercy Burn Center, Pittsburgh, PA; University of Pittsburgh Medical Center, Pittsburgh, PA; University of Pittsburgh Medical Center, Pittsburgh, PA

## Abstract

**Introduction:**

Peripheral vascular disease (PVD) impairs circulation and wound healing, potentially predisposing patients with lower extremity burns to worse outcomes. However, its impact on burn severity, hospital course, and mortality remains underexplored. This study evaluates the association between PVD and clinical outcomes in lower extremity burn patients.

**Methods:**

A retrospective review of the National Burn Repository (2013–2022) was conducted. Patients with lower extremity burns were identified, and those with a history of revascularization or amputation for PVD were compared to those without. Patient demographics, burn characteristics, and hospitalization data were analyzed using t-tests and chi-square tests. Multivariate logistic and linear regression assessed the association between PVD and mortality, length of stay, and reoperation for graft or flap loss.

**Results:**

Of 286 478 patients, 99 459 (34.7%) sustained lower extremity burns. 347 (0.4%) had a history of revascularization or amputation for PVD. Patients with PVD were significantly older than those without (64.6 vs. 38.0 years, *p*<.001) and had higher rates of comorbidities, including current smoking (*p*<.001), diabetes (*p*<.001), congestive heart failure (*p*<.001), and hypertension (*p*<.001). Despite a lower total body surface area (TBSA) burned (6.8% vs. 10.6%, *p*<.001), patients with PVD had longer hospital stays (16.4 vs. 12.2 days, *p*<.001). ICU length of stay (*p*=.149) and the number of operations required (*p*=.136) were similar between groups. On multivariate analysis, PVD was independently associated with increased mortality (OR 1.86, 95% CI 1.12–3.08, *p*=.017) and prolonged hospitalization (β = 3.58, 95% CI 1.73–5.43, *p*<.001) but was not associated with graft or flap loss requiring reoperation (*p*=.188).

**Conclusions:**

Patients with PVD experienced worse outcomes despite smaller burns. PVD is a significant risk factor for mortality and prolonged hospitalization in lower extremity burns, emphasizing the need for early recognition and tailored management in this high-risk population.

**Applicability of Research to Practice:**

This study highlights the need to incorporate peripheral vascular disease into burn risk assessments and perioperative planning. By recognizing PVD as an independent predictor of mortality and prolonged hospitalization, clinicians can apply more tailored monitoring, multidisciplinary involvement, and proactive management strategies to improve outcomes in this vulnerable patient population.

**Funding for the study:**

N/A.

